# Dental confidence and subjective well-being in young adults. The mediating role of self-esteem

**DOI:** 10.3389/froh.2025.1681685

**Published:** 2025-12-01

**Authors:** Beatrice Adriana Balgiu, Ruxandra Sfeatcu, Andreea Didilescu

**Affiliations:** 1 Department of Career and Educational Training, National University of Science and Technology Politehnica Bucharest, Bucharest, Romania; 2 Department of Oral Health and Community Dentistry, Faculty of Dentistry, “Carol Davila” University of Medicine and Pharmacy, Bucharest, Romania; 3 Department of Embriology and Microbiology, Faculty of Dentistry, “Carol Davila” University of Medicine and Pharmacy, Bucharest, Romania

**Keywords:** dental aesthetics, physical appearance, well-being, self-esteem, young adults

## Abstract

**Background:**

Dental aesthetic issues can negatively affect mental functioning and create barriers in social functioning in young individuals.

**Objective:**

This study aimed to analyze the role of self-esteem in the relationship between dental self-confidence and well-being in young Romanian adults and to test whether self-esteem functions as a mediator in this association.

**Methods:**

A cross-sectional study was employed, using a convenience sample of 775 respondents (Mean age = 21.74 years, *SD* = 3.40; 410 females) who were recruited to complete an online survey. This survey assessed dental self-confidence (scale from the Psychosocial Impact of Dental Aesthetics Questionnaire), self-esteem (Rosenberg Self-Esteem Scale), and subjective well-being via its cognitive and affective dimensions (Satisfaction with Life Scale and the Scale of Positive and Negative Experience). Partial least squares path modeling (PLS-SEM), a variance-based structural equation modeling method, was used to analyze the data.

**Results:**

The findings showed that the relationship between the influence of dental aesthetics and subjective well-being was partially mediated by self-esteem. Dental self-confidence positively impacted self-esteem (*β*=0.345), which in turn had a significant impact on well-being (β = 0.724). Together, self-esteem and dental self-confidence explained 59.9% of the variation in subjective well-being.

**Conclusions:**

The study concludes that young people who perceive themselves more positively regarding dental aesthetics tend to experience higher levels of well-being. The findings suggest that oral health campaigns to raise awareness of the psychosocial relevance of dental health could be beneficial.

## 1 Introduction

The importance of dental aesthetics in relation to body image, self-perception, and psychological well-being—especially in adolescents and young adults—is becoming more widely acknowledged.

Dental imperfections (e.g., misaligned teeth, discoloration, unattractive smile) can undermine self-confidence (1, 2), triggering embarrassment and social avoidance, which gradually erode self-esteem and reduce well-being (3). Furthermore, the stigmatization associated with dental problems may contribute to social exclusion (4), which in turn can lead to increased anxiety. One of the psychological concepts that is most impacted by dental appearance is self-esteem, commonly defined as a person's global sense of self-worth or value (5). Research indicates that high self-esteem serves as a protective factor against mental health difficulties and supports psychological resilience (6), whereas low self-esteem is linked to symptoms of anxiety and depression (7).

A psychosocial concept that has recently attracted attention in literature is dental self-confidence, defined as an individual's perception of the appearance and functionality of their own teeth (8–10). Concerns related to dental aesthetics, such as malocclusion or visible dental imperfections, have been associated with lower levels of self-confidence and heightened social anxiety (11–13).

To illustrate how dental appearance relates to self-esteem, the present study draws on two theoretical models. Thus, the present study is theoretically grounded in Harter's hierarchical model of self-esteem (2001) (14) and the Shavelson-Marsh model of self-concept (15). Harter posits that self-evaluations within specific domains, such as physical appearance, academic competence, and social acceptance, shape global self-esteem. Within this framework, dental self-confidence can be conceptualized as a subdomain of physical appearance, that contributes to one's overall self-worth. In the same vein, the Shavelson-Marsh model posits that self-concept is a multidimensional and hierarchical construct, where specific self-concepts (e.g., confidence in dental appearance) form broader categories (e.g., physical self-concept), which in turn feed into general self-concept and global self-esteem. These models collectively support the notion that self-esteem may act as a psychological mediator between specific physical self-perceptions and broader well-being outcomes. It can be argued that changes in dental self-confidence may indirectly influence subjective well-being, through their impact on self-esteem.

Several studies have confirmed the link between dental appearance and self-esteem. For instance, Venete et al. (16) found a positive correlation between dental confidence and self-esteem (r = 0.44), while similar patterns were reported by Stojilković et al. (12), and AlSagob et al. (9). Consistently with this, Dos Santos et al. reported that adolescents with lower global self-esteem experienced a more pronounced psychosocial impact related to dental appearance (17).

Clinical studies have confirmed these associations. Bahar et al., examining individuals undergoing orthodontic treatment, identified dental self-confidence—alongside PIDAQ dimensions including social impact and aesthetic concern—as significant predictors of self-esteem variation (11). Large-scale research further supports the generalizability of these patterns across cultural contexts. For instance, a study conducted in a sample of 962 Turkish university students, found that poor oral health was associated with lower self-esteem and negatively affected self-presentation and social behavior (18). Interventional research has also indicated improvements in psychological outcomes following aesthetic dental procedures. Studies have shown that orthodontic treatment, by improving dental alignment and smile aesthetics, leads to enhanced self-confidence, better social interactions, and higher life satisfaction—particularly in young populations (19, 20). As Kaur et al. argued, individuals often pursue orthodontic care not only for functional correction, but also for its anticipated psychosocial benefits, such as increased self-confidence, improved self-image, and reduced social anxiety (21). However, the psychological mechanisms through which the mentioned variables interact are underexplored.

Although the positive association between dental aesthetics and individual well-being is supported by previous research, the underlying psychological processes remain insufficiently explored. Building on this evidence, the present research investigates the relationship between confidence in dental appearance**,** subjective well-being, and self-esteem**.** The study population consisted of young Romanian adults, for whom dental aesthetics represent a central aspect of self-image, with potential impact on social functioning and well-being. To assess these relationships, we used a set of validated psychometric instruments. The study aimed to identify and clarify the psychological mechanisms through which dental confidence correlates with subjective well-being, paying special attention to the mediating role of self-esteem. Unlike previous studies (9, 12, 16, 17) that mainly examined the impact of dental aesthetics on self-esteem, the present research investigates whether self-esteem mediates the relationship between perceived dental aesthetics and subjective well-being. Investigating the mediating role of self-esteem, the research attempts to identify the psychological mechanism through which self-esteem influences the well-being and implicitly the quality of life of young people.

Hypotheses. Based on the literature the following hypotheses were formulated:

H1: Dental self-confidence, self-esteem, and subjective well-being are positively correlated.

H2: Self-esteem mediates the relationship between dental self-confidence and subjective well-being.

## 2 Methods

### 2.1 Participants and procedure

The study employed a cross-sectional design and was conducted on a sample of young adults in Romania. A snowball sampling method was used. Participants were recruited via social media platforms (e.g., Facebook and WhatsApp) and were provided with a hyperlink to a Google Forms survey that included a series of standardized assessment tools. The “initial seeds” were university students from the Faculty of Medical Engineering (National University of Science and Technology Politehnica Bucharest), who were invited to share the survey link with their peers via social media platforms. Recruitment proceeded in four successive waves as participants further distributed the link within their networks. The population primarily consisted of young adults from both urban and rural areas across Romania, although urban participants were overrepresented. To reduce sampling bias, participation was anonymous (22). Demographic information was collected to monitor representation, especially across gender and education. The eligibility criteria required individuals to be aged between 18 and 35 years, possess Romanian as their primary language, and have attended a dental appointment at least once prior to the completion of the assessment tools. Incomplete responses, non-informed consent, and age outside the target range of 18–35 years were among the exclusion criteria. Participants were offered comprehensive guidance on how to accurately complete the questionnaires, which also included information pertaining to informed consent. The average time to complete the questionnaire was approximately 8–9 min. At the beginning of the survey, a short explanatory text was provided outlining the purpose of the study, emphasizing that participation involved no risks or rewards.

Data collection was carried out during the period 17.01.-21.05.2023. Given that the survey was distributed online using a snowball sampling approach, the exact number of individuals who received the invitation to complete it cannot be determined; therefore the response rate remains unknown.

The required sample size was calculated according to the recommendations for structural equation modeling. The minimum recommended sample size of 223 respondents was calculated based on an expected effect size of 0.30, a desired statistical power level of 0.95, a probability level of 0.05, 5 latent variables, and 29 observable variables.

### 2.2 Measures

1. *Dental Self-Confidence Scale (DSCS)* is a subscale derived from the PIDAQ (23), consisting of six items that assess the emotional impact of dental aesthetics on the individual. Responses are rated on a 5-point Likert scale ranging from 0 (*not at all*) to 4 (*very strongly*) (e.g., *I am proud of my teeth*; *I like to show my teeth*). The Romanian version validated on the adult population was used (24). In the present study, DSCS items were not reverse-coded. Factor analysis conducted for this scale yielded satisfactory fit indices: *χ*²/df = 2.04; CFI = 0.99; RMSEA = 0.067; SRMR = 0.010, and α and ω coefficients have values of 0.91.
2. *Scale of Positive and Negative Experience (SPANE)* consists of 12 items designed to assess the frequency of emotional experiences. Six items describe positive feelings (SPANE-P), while the other six describe negative feelings (SPANE-N). Responses are recorded on a 5-point scale ranging from 1 (*very rarely or never*) to 5 (*very frequently or always*) (e.g., “I felt happy”, “I felt sad”). Each subscale includes three general emotional descriptors (e.g., *positive*, *pleasant*, *negative*) and three that are more specific (e.g., *happy*, *joyful*, *sad*). The inclusion of both general and specific emotional terms allows the instrument to capture a broader spectrum of emotional experiences, ranging from subtle to intense (25). For this study, we used the Romanian-validated version of the scale (26). The CFA conducted on the present sample supported the scale structure, with adequate model fit indices: *χ*²/df = 3.38; CFI = 0.97; RMSEA = 0.056; SRMR = 0.034; and Cronbach's alpha (α) and McDonald's omega (ω) for SPANE-P have values of 0.90 and 0.87 and, respectively, 0.88 for SPANE-N.
3. *Satisfaction with Life Scale (SWLS)* (Diener et al., 1985) is one of the most widely used tools for measuring overall life satisfaction, reflecting the cognitive dimension of subjective well-being (27). The scale comprises 5 items rated on a on a 7-point Likert scale ranging from 1 (*strongly disagree*) to 7 (*strongly agree*) (Sample item: *In most ways my life is close to my ideal*). In Romania, the scale has been validated across various population groups and has demonstrated solid psychometric properties (28, 29). In the current study, the scale again showed high internal consistency, with α = 0.86 and *ω*=0.86. Confirmatory factor analysis (CFA) indicated good model fit: *χ*²/df = 1.18; CFI = 0.99; RMSEA = 0.016; SRMR = 0.026.
4. *Rosenberg Self-Esteem Scale (RSES)* (Rosenberg, 1989) is a widely used instrument designed to measure global self-esteem across various age groups (30). It comprises 10 statements rated on a 4-point Likert scale, from 1 (*strongly disagree*) to 4 (*strongly agree*). Half of the items are negatively worded and require reverse scoring (e.g., *I wish I had more respect for myself*). The overall self-esteem score is calculated by summing all item responses, with higher values indicating a stronger sense of self-worth. In this study, we employed the Romanian-adapted version of the scale (31). Internal consistency indices confirmed the reliability of the scale in our sample, with α = 0.86 and ω = 0.87. Confirmatory factor analysis supported the unidimensional structure of the scale, with the following model fit indices: *χ*²/df = 2.96; CFI = 0.98; RMSEA = 0.050; SRMR = 0.037.
  The complete list of items from all instruments used is presented in Supplementary Table SM1. In this text, only illustrative items are presented for brevity.
5. *A short questionnaire concerning sociodemographic data.* The information collected includes data on: (i) self-identified gender, (ii) age, (iii) studies (primary, secondary, university and post-university), (iv) area of residence (urban vs. rural), (v) employment sector (public, private, or other), and (vi) geographical region.

### 2.3 Data analysis strategy

Descriptive (means and standard deviations), correlational analyses, and structural equation modeling using the PLS-SEM were conducted to examine the relationships between variables. The latter was selected given the exploratory purpose of the study, with the aim of identifying and validating the mediating role of self-esteem in the proposed model.

Several indices were used to evaluate the quality and fit of the model, including:

Dijkstra–Henseler's rho (*ρ*A), Jöreskog's rho (*ρ*c), Cronbach's alpha, Variance Inflation Factors (VIF), Average Variance Extracted (AVE), Standardized Root Mean Square Residual (SRMR), Heterotrait–Monotrait Ratio (HTMT), and R² values for explained variance. According to general recommendations, cut-off values for *ρ*A, *ρ*c, and Cronbach's alpha should exceed 0.70 to indicate acceptable reliability (32). The AVE should be at least 0.50 to support convergent validity, and SRMR values below 0.080 are indicative of a good model fit. For discriminant validity, HTMT values should remain below 0.90, or more conservatively, below 0.85 (33). In addition, to demonstrate the absence of multicollinearity, VIF values should be below 5.00 (34). All inferential analyses were performed at a significant level of *p* < 0.001. To assess the statistical significance of the model parameters, a bootstrapping procedure with 5.000 resamples was conducted, in line with the recommendations of Henseler et al. (33).

All data analyses were performed using SPSS24 (IBM, Corp. Armonk, NY, USA) and ADANCO 2.4.0 (University of Twente, Netherlands).

### 2.4 Ethical approval

The informed consent was obtained from all participants involved in the study. This study was approved by the Ethical Commission of the “Carol Davila” University of Medicine and Pharmacy, Bucharest (Protocol No. 1141/13.01.2023).

The study was conducted in full accordance with ethical principles, including the World Medical Association Declaration of Helsinki from 1975 as revised in 2013.

## 3 Results

### 3.1 Sociodemographic characteristics of the sample

The final sample consisted of 775 young adults (Mean age = 21.74 years; *SD* = 3.40), of whom 410 (52.9%) were females (Mean age = 22.12; *SD* = 3.46) and 365 (47.09%) males (Mean age = 21.05; *SD* = 3.18) reflecting a balanced gender distribution (Table 1). In terms of geographic background, most participants (82.79%; *N* = 640) reported living in urban areas, while 17.21% (*N* = 133) resided in rural areas. Regarding educational attainment, participants exhibited a diverse range of academic backgrounds: 52.4% had completed secondary education, 44.0% were enrolled in or had completed university studies, and 3.6% held post-graduate degrees. The sample also varied occupationally. A portion of respondents (22.76%; *N* = 176) reported working in the public sector, while 20.69% (*N* = 160) were employed in private companies. A smaller segment identified as freelancers (12.03%; *N* = 93), and the largest group (44.50%; *N* = 344) indicated that they were unemployed at the time of data collection. Most participants originated from the capital area and its districts (Bucharest-Ilfov, 53.16%), followed by the South-East (20.64%) and North-East (15.50%). The remaining participants were from other regions of the country (10.70%), with the western region being the least represented, accounting for only 2.32% of the sample.

**Table 1:** Socio-demographic characteristics of the sample.

Variables	*N* = 775	
Age	18–35 years	Mean_age:_ 21.74; *SD* = 3.40
Gender	Males	47.09
	Females	52.91
Residence	Urban	82.79
	Rural	17.21
Education	High school	52.4
	Undergraduate studies	44.0
	Graduate degree	3.6
Work sector	Public	22.76
	Private	20.69
	Freelance	12.05
	Unemployed	44.5
Geographical region	Bucharest-Ilfov	53.16
	South-East	20.64
	North-East	15.50
	Other regions	10.7

Results are given as percentages; *SD*-standard deviation.

### 3.2 Common method bias (CMB)

The possibility of respondent social desirability bias was assessed in two ways. Initially, Harman's single-factor test (35) was conducted via exploratory factor analysis (EFA) revealing that factorial solution illustrated 5 distinct factors greater than 1 (KMO = 0.943; Bartlett's test of sphericity = 16,039.105; *p* < 0.001). The first factor captured 34.48% of the data variance and scored below the 50% recommended threshold (36). Subsequently, a model with one latent factor was tested through confirmatory factor analysis (CFA), in which case poor values of the fit indices were observed: *χ*² = 7,898.389; *χ*²/df = 15.95; CFI = 0.531; TLI = 0.500; RMSEA = 0.139 (0.136–0.142); SRMR = 0.121. These results show that there is no significant evidence of CMB in this present study.

### 3.3 Descriptive and correlational analysis

Table 2 presents the descriptive and the correlational analysis of the investigated variables: dental self-confidence, self-esteem, and subjective well-being. The latter was computed following Diener's model (2010) (25), by combining the scores of the SWLS and SPANE. Prior to aggregation, both scales were standardized (z-scores) to account for differences in response format. The average score for dental self-confidence was M = 16.553 (*SD* = 5.325). Statistical comparisons between studies conducted in different cultural contexts should be considered with caution to contextualize our findings. These comparisons are illustrative and do not imply statistical significance, as no formal tests were conducted across samples. Therefore, the present sample showed higher levels of dental self-confidence than young Arab women (M = 8.5) (9), lower than mixed-gender samples from Valencia (M ≥ 22) (16), and approximately equivalent with Serbian youth (M = 14) (12).

**Table 2:** The descriptive and correlational analysis of the variables.

Variables	Mean	*SD*	Min-max	1	2
1. Dental self-confidence	16.553	5.325	0–24	–	
2. Self-esteem	31.122	5.866	12–40	0.301***	–
3. Well-being	47.772	9.687	11–65	0.333***	0.683***

*** *p* < 0.001.

For self-esteem, M = 31.122 (*SD* = 5.866), suggesting that most participants perceived themselves as having moderate global self-esteem. The average self-esteem score was slightly lower than the scores obtained in the Western population (where the average is 39 for men and 40 for women) (16). In the case of subjective well-being, M = 47.772 (*SD* = 9.687) indicated that participants generally reported a positive level of subjective well-being, consistent with the expectation that well-being would be positively correlated with both dental confidence and self-esteem. The mean well-being score is slightly higher than those observed in other young adult samples using SWLS and SPANE (25, 29). These comparisons should be interpreted with caution, as they are intended only to illustrate potential cultural variations rather than to establish significant differences. The comparisons suggest that our sample broadly reflects the psychological patterns documented in young adult populations, providing a valid context for subsequent analyses.

For the Dental Self-Confidence Scale, the highest mean was recorded for “*I am proud of my teeth”* (M = 2.839; *SD* = 0.91), while the lowest was for “*My teeth are attractive to others*” (M = 2.631; *SD* = 1.07), suggesting a slightly higher satisfaction with one's own teeth than with perceived social attractiveness. Within the Self-Esteem Scale, positively worded items such as “*I feel that I have a number of good qualities”* (M = 3.498; *SD* = 0.687) are in contrast to reversed items like “*All in all, I am inclined to feel that I am a failure*” (M = 1.509; *SD* = 0.834), reflecting a moderate-to-positive global self-evaluation. Similar patterns were observed for the well-being measures, where positive experiences (e.g., “*I felt happy”*, M = 3.886; *SD* = 0.983, and “*I felt good*”, M = 3.846; *SD* = 0.943) received higher scores than negative ones (“*I felt sad”*, M = 2.723; *SD* = 1.140). A table with the average scores for each item from the instruments used can be found in Supplementary material SM1.

All correlations between the variables were statistically significant (*p* < 0.001). Dental self-confidence (DSC) was positively correlated with self-esteem (r = 0.301; *p* < 0.001) and well-being (r = 0.333; *p* < 0.001). Additionally, self-esteem was positively correlated with well-being (r = 0.683; *p* < 0.001). These results suggest that higher levels of dental self-confidence and self-esteem are associated with higher levels of subjective well-being, confirming hypothesis H1.

### 3.4 Evaluation of the PLS-SEM model indices

In the second stage, a structural equation model was developed and evaluated using the aforementioned indices. An initial model was tested, which revealed low factor loadings for two SPANE items (items 10 and 12 with factor loadings of 0.321 and 0.400, respectively) and one item from the RSES (item 3—factor loading: 0.272). Following the recommendations of Hair et al. (34), these items were removed to improve convergent validity. The core constructs remain intact despite the removal of these items. After removal, the Average Variance Extracted (AVE) increased from 0.410 to 0.450 for self-esteem (SE) and to 0.452 for well-being (WB), while Composite Reliability remained above the minimum acceptable threshold.

Further analysis of model fit indices indicated that all constructs demonstrated strong internal consistency and satisfactory measurement quality. The composite reliability (*ρ*c) values ranged from 0.876 to 0.924, and Cronbach's alpha (*α*) values ranged from 0.880 to 0.923, exceeding the recommended threshold of 0.70 (34) (Table 3). Variance Inflation Factor (VIF) values were below the critical limit of 5.00, falling within a range of 1.442–4.074, suggesting no multicollinearity issues. The standardized factor loadings ranged from 0.51 to 0.91, all above the minimum acceptable value of 0.50, supporting the presence of convergent validity. AVE for dental self-confidence was over 0.50 and has a value over 0.45 for self-esteem and well-being. However, according to literature an AVE value below 0.50 is not a problem if *ρ*c is 0.70 (37).

**Table 3:** Evaluation indices of the model (reliability, convergent validity).

Variables	*ρ*_A_ (>0.70)	*ρ*_c_ (>0.70)	α (>0.70)	Loadings (interval)	VIF (<5.00)	AVE (>0.50)
1. DSC	0.931	0.922	0.921	0.597–0.901	1.847–4.074	0.667
2. SE	0.895	0.876	0.880	0.543–0.883	1.540–3.419	0.450
3. WB	0.929	0.924	0.923	0.498–0.880	1.442–3.364	0.452

DSC, dental self-confidence; SE, self-esteem; WB, well-being; ρA, reliability rho; ρc, composite reliability; α, Cronbach's alpha; VIF, variance inflation factors, AVE, average variance extracted.

All HTMT (Heterotrait–Monotrait ratio) values were below the conservative threshold of 0.85, ranging from 0.331 to 0.752, which suggests that each latent construct is empirically distinct from the others (Table 4). The SRMR was 0.0772. These findings collectively support the reliability and validity of the measurement model.

**Table 4:** Discriminant validity: heterotrait-monotrait ratio of correlations (HTMT).

Constructs	1	2
1. Dental self-confidence		
2. Self-esteem	0.331	
3. Well-being	0.365	0.752

### 3.5 The analysis of the model

Dental self-confidence had a statistically significant direct effect on both self-esteem (β = 0.345; *p* < 0.001) and well-being (β = 0.121; *p* < 0.001). Furthermore, self-esteem significantly predicted well-being (β = 0.724; *p* < 0.001), indicating a strong direct influence (Table 5). Therefore, self-esteem has a significant impact on subjective well-being. This supports the mediating role of self-esteem in the relationship between dental self-confidence and well-being. The indirect effect of dental self-confidence on subjective well-being via self-esteem was also significant (β = 0.249; *p* < 0.001), highlighting a substantial mediating mechanism. Thus, hypothesis H2 is accepted.

**Table 5:** Direct, indirect, and total effects in the structural model.

Direct effect	β	SE	t-value	*p*-value<
DSC -> WB	0.121	0.03	3.758	0.001
DSC -> SE	0.345	0.02	9.512	0.001
SE -> WB	0.724	0.03	29.358	0.001
Indirect effect				
DSC-> WB	0.249	0.02	8.889	0.001
Total effect				
DSC -> WB	0.370	0.02	10.544	0.001

*Β*, standardized path coefficient; SE, standard error; *p*, probability value; significance threshold set at *p* < 0.001.

In terms of explained variance, the structural model revealed that dental self-confidence accounted for 11.9% of the variance in self-esteem (R² = 0.119), indicating a modest but significant contribution to the development of global self-esteem.

**Figure 1:**
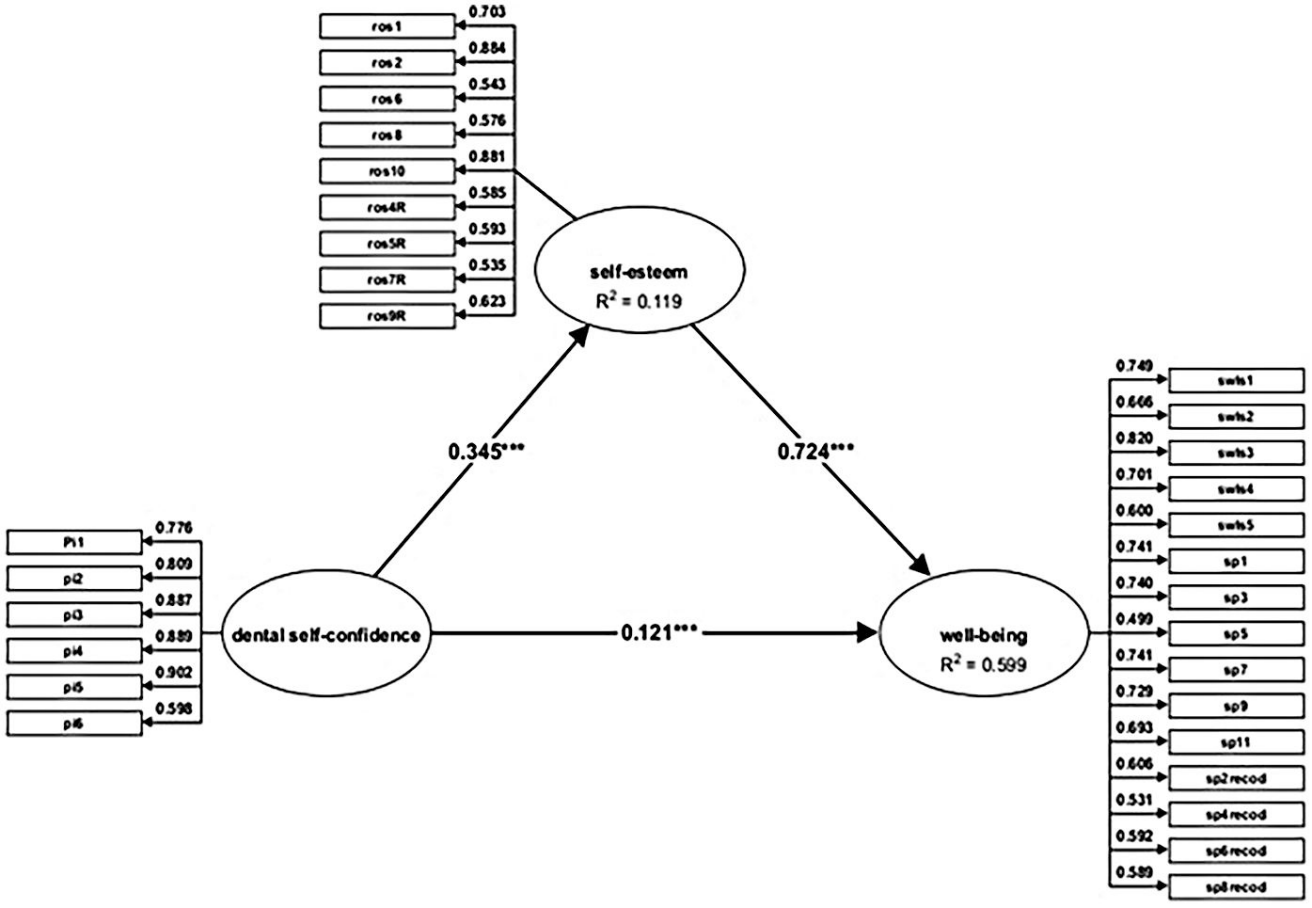
The mediating effect of self-esteem in the relationship between dental self-confidence and well-being (β—path coefficients and R^2^ values). ***p < 0.001.

Notably, the combination of dental self-confidence and self-esteem explained 59.9% of the variance in subjective well-being (R² = 0.599), which is considered a substantial effect according to established SEM benchmarks (Hair et al., 2019) (34). This high level of explained variance suggests that both perceived dental appearance and self-evaluations of worth play major roles in shaping emotional and cognitive well-being in young adults (Figure 1).

## 4 Discussion and conclusions

This study aimed to investigate the relationship between dental self-confidence and subjective well-being, with a particular focus on the mediating role of self-esteem. The findings offer strong empirical support for the hypothesized model and are consistent with theoretical assumptions regarding the mechanisms linking physical self-perception to broader psychological functioning. As expected, dental self-confidence was significantly associated with higher levels of self-esteem. This result aligns with prior research indicating that perceived physical attractiveness, including specific features like dental aesthetics, contributes to a more positive global self-concept (16, 17, 38). In comparison with other studies conducted on young adult populations, our findings are consistent with those reported by Stojilković et al. (12), who found a negative correlation between dental dissatisfaction and self-esteem (r = −0.316) among Serbian university students, and Venete et al. (16), who identified a positive correlation of dental self-confidence and self-esteem (r = 0.357). These values closely align with the correlation observed in our sample between dental confidence and self-esteem (r = 0.301). Similarly, the research conducted by Bahar et al. (11), found that dental self-confidence significantly predicted self-esteem and, indirectly, the psychosocial dimension of quality of life, as indicated by regression analyses, in young patients undergoing orthodontic treatment. In the same direction, the study conducted by Yüzer Alsaç et al. (18) reported a significant negative association between perceived oral health and self-esteem, as well as difficulties related to self-presentation and social integration, among Turkish students. Collectively, these findings reinforce the notion that dental self-perceptions exert an influence on psychological self-assessment in this age group. From a sociocognitive perspective, self-confidence in one's appearance—particularly in visible domains such as the smile—may enhance self-perceived social acceptability, thereby reinforcing self-worth (15).

Furthermore, self-esteem emerged as a robust predictor of subjective well-being, confirming its established role as a core determinant of mental health and life satisfaction (39). Individuals with higher self-esteem tend to evaluate their lives positively and experience higher levels of emotional well-being (40).

The finding that dental self-evaluation accounts for 11.9% of the variance in self-esteem, and that together they explain nearly 60% of the variance in subjective well-being (R²=0.599), suggests a potentially strong association between dental self-image and subjective well-being among Romanian youth. Critically, the results supported a partial mediation model, in which self-esteem explained a substantial portion of the effect of dental self-confidence on well-being, but a small direct path remained significant (40.1%). This means that, although self-esteem mediates a considerable part of the influence of dental confidence on well-being (a significant indirect effect), a residual direct effect of dental self-confidence on subjective well-being remains even after accounting for the role of self-esteem.

Therefore, beyond its impact on self-esteem, dental confidence may be associated with well-being through additional pathways that are not fully explained by self-worth. This result suggests that while improved perceptions of one's dental appearance enhance well-being indirectly through their impact on self-esteem, some aspects of this relationship are not fully accounted for by self-worth alone. It is possible that dental self-confidence also contributes directly to emotional comfort in social interactions, reduced appearance-related anxiety, and increased willingness to engage in positive experiences, all of which may influence subjective well-being independently of self-esteem (11, 19).

The relevance of self-esteem to overall well-being may be further explained by its association with social anxiety. Young individuals with dental aesthetic impairments are more likely to experience social anxiety, avoid eye contact, and exhibit difficulties in smiling in public (3, 41, 42). This relationship is, of course, also shaped by prevailing social norms. Research indicates that perceptions of facial aesthetics—particularly dental alignment—are influenced by cultural standards (43). Romanian standards regarding dental aesthetics seem to be shaped by increasing exposure to Western ideals of the “perfect smile.” (e.g., white, straight teeth and an attractive smile). It is well established that Western societies tend to prioritize physical attractiveness, including dental aesthetics, as a pathway to both social and personal success (44). For example, studies on Romanian patients show that they place importance on tooth colour and smile aesthetics (45). However, these norms are tempered by structural constraints, such as lower public coverage of dental offices and financial barriers to accessibility (46). This makes dental aesthetics for young Romanians seem less of a priority compared to Western Europe, where cosmetic dental improvements are more expected and funded (47). These cultural differences are also reflected in our results: as shown in the descriptive data, the present sample reported higher levels of dental self-confidence than predominantly collectivist Arab groups, but lower scores than Spanish youth, who represent a Western, highly individualistic context. This pattern seems to suggest that young Romanian adults are influenced by both collective traditions and Western pressures. Further research is needed to explore how cultural context moderates the relationship between the analysed variables.

The findings of this study contribute to the growing body of literature emphasizing the psychological relevance of dental and facial aesthetics, particularly in young adults, where self-image is important (11, 48). The study's results show that dental confidence is not just a surface-level concern; it has real effects on emotional health and quality of life. The results can be used to support interventions aimed at improving the appearance of teeth (for example, orthodontic or cosmetic treatments like teeth whitening) not only for their practical benefits but also for their positive psychological effects, which are enhanced by increased self-esteem. Simultaneously, psychological treatments that focus on boosting self-esteem can have positively affect changes in body image, such as those related to the appearance of teeth.

### 4.1 Study limitations and future research directions

One limitation of this study is its cross-sectional design, which does not allow for testing of causal relationships. Future research should address these limitations by using longitudinal assessments. An important direction that should be tested in future research is the extension of the investigation to clinical populations or adolescents, where the impact of body image is often amplified. Last but not least, the study focused solely on self-esteem as a mediator. It is recommended that other relevant psychological factors (e.g., social anxiety, global body image satisfaction, oral health values, or oral health inequalities) be explored in future studies. The analysis also did not consider control variables that could simultaneously influence both self-esteem and subjective well-being, including socioeconomic and dental health status. Integrating such factors in future research would allow for a better delineation of the specific effects of dental confidence and would increase the robustness of explanatory models.

Although the use of self-report scales is common in psychological studies, especially those that evaluate participants’ perceptions (49), it also makes the results susceptible to method biases. An additional limitation stems from the use of snowball sampling which, although practical and cost-effective, presents risks of bias, increasing the likelihood of recruiting participants with similar characteristics thereby limiting the generalizability of the findings (50). In the context of this study, it is possible that the sample is over-represented by young people from urban environments (82.79% of participants) or from connected social networks, which could limit the generalizability of the findings to other populations, such as young people from rural areas or from more diverse groups. The impact of dental confidence on self-esteem and overall well-being may differ between urban and rural areas. Because they are more exposed to Western aesthetic standards, young people in urban areas are more concerned with physical attractiveness, beauty, and dental appearance. The effect of dental confidence on self-esteem and subjective well-being may be lessened or recalculated in rural areas due to limited access to dental services and disparate aesthetic standards. Future studies should examine these contextual differences in comparison or use more balanced urban-rural samples in order to gain a more nuanced understanding of the role of socio-geographical factors.

Another methodological limitation concerns the adjustments made to the psychometric scales. To improve convergent validity, two items from SPANE and one from RSES were removed; although this procedure is acceptable and supported by previous literature (34, 51), it may limit direct comparability with studies employing the full versions of these instruments in structural equation modeling. However, our findings suggest that this difference is mainly quantitative, reflected in the values of the path coefficients, rather than qualitative. The basic significance of the constructs was preserved, as the relationships between variables remained stable, and self-esteem continued to function as a mediator in the model. The impact of these adjustments is primarily quantitative rather than qualitative and does not undermine the theoretical interpretation of constructs.

The AVE values for self-esteem and well-being can constitute a minor limitation as long as their values are slightly below the conventional threshold of 0.50. However, the high composite reliability supports the adequacy of these constructs (34).

### 4.2 The theoretical and practical implications of the study are as follows

First, by focusing on the role of self-esteem as a mediator, the study strengthens cognitive views on self-identity and advances the idea of how psychological health and physical appearance are related. The results could be used to improve more completely positive psychology models, which support the idea that being happy with oneself in certain areas can have a positive effect on your overall health. Second, these results may show dental professionals that making teeth look better can boost patients’ confidence and, as a result, may increase perceptions of quality of life, although longitudinal and interventional studies are needed to confirm the results. Clinical psychologists and counselors can include worries about dental image in treatments that are meant to boost self-esteem, especially for teens who are socially anxious, ashamed of their bodies, or emotionally vulnerable. From this perspective, dental professionals should be aware of the patient's behaviors of avoiding eye contact, anxiety, and embarrassment when smiling and encourage the expression of fears and expectations related to dental aesthetics. In this regard, we propose the administration of the Dental Self-Confidence Scale (DSCS) as a psychological screening tool in the initial patient assessment given that it has the advantage of a short scale. In the office, the approach must be empathetic, personalized and oriented towards the patient's psychology. It is even necessary to have a close interdisciplinary collaboration between the dentist and the psychologist, thus becoming a recommended approach, with obvious benefits for the patient's mental health and quality of life.

From a theoretical perspective, the model supports and enriches the biopsychosocial framework, suggesting that dental aesthetics influence well-being via psychological pathways, particularly self-esteem. This highlights the importance of integrating psychological screening and support into dental and orthodontic care, especially for individuals expressing dissatisfaction with their dental appearance. Preventive interventions that enhance self-esteem may serve as protective factors against the negative psychological impact of dental concerns. Additionally, these findings may inform public health messaging by promoting oral health not only as a matter of hygiene or aesthetics, but also as a determinant of broader psychosocial well-being.

## Data Availability

The raw data supporting the conclusions of this article will be made available by the authors, without undue reservation.
