# Supplementary material for: Dental confidence and subjective well-being in young adults. The mediating role of self-esteem
Source: Front Oral Health. 2025 Dec 1;6:1681685. doi: 10.3389/froh.2025.1681685 (PMC12702907; doi:10.3389/froh.2025.1681685)
Supplement: Supplementary file 1 [file Table1.docx]

**Supplementary Table SM1.** *Descriptive statistics for all items of the scales used in the study (DSCS, SPANE, SWLS, RSES)*

| Measures | Items | Mean | *SD* |
| --- | --- | --- | --- |
| DSCS | 1. I am proud of my teeth | 2.839 | 0.910 |
|  | 2. I like to show my teeth when I smile | 2.747 | 1.227 |
|  | 3. I am pleased when I see my teeth in the mirror | 2.790 | 0.992 |
|  | 4. My teeth are attractive to others | 2.631 | 1.068 |
|  | 5. I am satisfied with the appearance of my teeth | 2.796 | 0.975 |
|  | 6. I find my tooth position to be very nice | 2.747 | 1.124 |
| SPANE | 1. Positive feelings | 3.736 | 0.967 |
|  | 2. Negative feelings | 2.804 | 1.110 |
|  | 3. I felt good | 3.846 | 0.943 |
|  | 4. I felt bad | 2.627 | 1.088 |
|  | 5. Pleasant feelings | 3.697 | 0.950 |
|  | 6. Unpleasant feelings | 2.483 | 1.051 |
|  | 7. I felt happy | 3.886 | 0.983 |
|  | 8. I felt sad | 2.723 | 1.140 |
|  | 9. I felt afraid | 3.873 | 0.935 |
|  | 10. I felt joyful | 2.615 | 1.297 |
|  | 11. I felt angry | 3.732 | 1.026 |
|  | 12. I felt contented | 3.346 | 1.243 |
| SWLS | 1. In most ways my life is close to my ideal | 4.566 | 1.437 |
|  | 2. The conditions of my life are excellent | 5.094 | 1.376 |
|  | 3. I am satisfied with my life | 5.283 | 1.379 |
|  | 4. So far I have gotten the important things I want in life | 5.410 | 1.407 |
|  | 5. If I could live my life over, I would change almost nothing | 4.646 | 1.781 |
| RSES | 1. I feel that I'm a person of worth, at least on an equal plane with others | 3.351 | 0.790 |
|  | 2. On the whole, I am satisfied with myself | 3.218 | 0.795 |
|  | 3. I wish I could have more respect for myself* | 3.022 | 0.995 |
|  | 4. I certainly feel useless at times* | 2.066 | 1.006 |
|  | 5. At times, I think I am no good at all* | 2.049 | 1.019 |
|  | 6. I feel that I have a number of good qualities | 3.498 | 0.687 |
|  | 7. All in all, I am inclined to feel that I am a failure* | 1.509 | 0.834 |
|  | 8. I am able to do things as well as most other people | 3.375 | 0.757 |
|  | 9. I feel I do not have much to be proud of* | 1.800 | 0.924 |
|  | 10. I take a positive attitude toward myself | 3.126 | 0.857 |

*Note*: *SD*-Standard deviation; * items with asterisks are reverse score
